# Institutional Needs

**Published:** 1916-01-01

**Authors:** 


### Institutional Needs.

THE ALLSOPP'S VEGETABLE AND FRUIT SLICER.

(Mr. J. C. Allsopp, 87 Gertrude Road, West Bridgford, Nottingham.)

A highly ingenious machine for slicing oranges, lemons, cucumbers, beans, and all kinds of fruit is made by Mr. J. C. Allsopp, 87 Gertrude Road, West Bridgford, Nottingham. Perhaps its virtues are most apparent when much rapid and even cutting is required, as, for instance, in the case of marmalade-making. Its retail price is 20s. and 17s. 6d., while it can be fitted with a grooved wheel for electric, and fast and loose pulleys for steam power at a cost of 35s. and 45s. respectively. While the Allsopp machine will cut 2 lb. per minute, it is also easy to clean, and this should be sufficient to establish its claim for institutional purposes among those who have not already proved its value.

